# 

**Published:** 1837-10-01

**Authors:** 


### BIBLIOGRAPHICAL RECORD.

1. The Works of John Hunter, F.R.S. with Notes. Edited by JAMES F. PALMER, &c. &c. In four vols. Vol. III. With Plates. July, 1837.

---

2. A History of British Quadrupeds. By THOMAS BELL, F.R.S. Parts X. & XI. price 2s. 6d. each.

---

3. A History of British Birds. By WILLIAM YARRELL, Esq. Secretary to the Zoological Society. Parts I. & II. price 2s. 6d. each. Illustrated with wood-cuts. Van Voorst, 1837.

☞ *Very excellent.*

---

4. Medical Relief for the Labouring Classes; on the Principle of Mutual Insurance. By H. W. RUMSEY, Surgeon. 8vo. pp. 69. London, 1837.

---

5. Observations (in Manuscript) on Cholera. By Dr. JOHN ADAMSON, of New York.

---

6. The Spas of Germany. By the Author of St. Petersburgh. 2 vols. 8vo. with numerous wood-cuts. Colburn, July 1837. (From the Publisher.)

---

7. The Philosophy of the Eye, being a familiar exposition of the Mechanism and of the Phenomena of Vision, with a View to the Evidence of Design. By JOHN WALKER. With numerous Illustrations. One Volume. 8vo. pp. 300. Knight, 1837.

---

8. On a New Mode of Treatment employed in the Cure of various forms of Ulcer and Granulating Wounds. By FREDERICK C. SKEY, F.R.S. Assistant Surgeon to St. Bartholomew's Hospital, &c. 8vo. pp. 85. Longman and Co. July, 1837.

---

9. Essay on the Classification of the Insane. By M. ALLEN, M.D. 8vo. pp. 208, with an Appendix, pp. 146. Taylor, London, 1837.

---

10. Letters to the Right Honourable the Lord Provost and Town-Council of Edinburgh. By Dr. KNOX.

---

11. On the Nature and Treatment of the Diseases of the Heart; with some New Views on the Physiology of the Circulation. By JAMES WARDROP, M.D. Part the First, with Plates. 8vo. Churchill, London, July, 1837.

---

12. An Exposition of the Signs and Symptoms of Pregnancy—the Period of Human Gestation—and the Signs of Delivery. By W. F. MONTGOMERY, A.M. M.D. &c. Sherwood, London, 1837.

---

13. Dental Practice, or Observations, &c. By JOHN GRAY, Surgeon-Dentist. Duodecimo, 1837.

---

14. Observations on the Topography, Climate, and Prevalent Diseases of the Island of Jersey, the Result of Meteorological Observation, &c. By GEO. S. HOOPER, M.D. 8vo. Whittaker, London, July, 1837.

---

15. Continental and British Medical Review—July, August and September. Conducted by Dr. Bureaud Riofrey.

16. The Cyclopædia of Anatomy and Physiology. Edited by R. B. Todd, M.D. Part XI. Containing Eye—Face—Fascia—Fat—Femoral Artery—Fibrine—Fibro-Cartilage—Fibrous Tissue—Fibular Artery—Fifth Pair of Nerves. Illustrated with numerous Engravings. Price 5s. August, 1837.

---

17. Plan for Regulating Medico-Parochial Attendance; also Observations on Self-supporting Dispensaries and Union-Infirmaries. By John Charlton Yeatman. Fourth Edition enlarged. Longman and Co. 1837.

---

18. Surgical Observations on Tumours, with Cases and Operations. By J. C. Warren, M.D. Professor of Anatomy and Surgery in Harvard University, Surgeon to the Massachusett's General Hospital. 8vo. pp. 608, with Plates. Boston, 1837.

---

19. The Physician's Vademecum; or a Manual of the Principles and Practice of Physic, &c. &c. &c. By Robert Hooper, M.D. New Edition, considerably enlarged and improved. By Michael Ryan, M.D. 8vo. 1837.

---

20. A Manual of Percussion and Auscultation, as employed in the Diagnosis of Diseases of the Chest and Abdomen, 32mo. By Dr. Spillan.

---

21. An Examination of Phrenology; in Two Lectures, delivered to the Students of the Columbian College, &c. By Thomas Sewall, M.D. with a Plate. Washington City, 1837.

---

22. An Experimental Essay on the Relative Physiological and Medicinal Properties of Iodine and its Compounds, being the Harveian Prize Dissertation for 1837. By Charles Cogswell, M.D. A.B. &c. &c. Edinburgh, 1837.

---

23. Notice of Patents granted to Joseph Amesbury, of Burton Crescent, for certain Apparatus used in the Treatment of Stiffness, Weakness, &c.

---

24. The Phrenological Journal, No. 53. Sept. 1837, 2s. 6d. Maclachlan and Stewart, 1837.

---

25. The Dublin Journal of Medical Science, &c. for September, 1837.

26. Advice on the Care of the Teeth. By Edwin Saunders, Dentist; Fellow of the Medico-Botanical Society, &c. 12th Edition, 1837.

---

27. Inaugural Dissertation on the Physiology and Pathology of the Brain; being an Attempt to ascertain what portions of that Organ are more immediately connected with Motion, Sensation, Intelligence, &c. By John Hughes Bennett, &c. Edinb. 1817.
☞ *We may notice this hereafter.*

---

28. A Medico-Topographical, Geological, and Statistical Sketch of Bolton and its Vicinity. By Jas. Black, M.D. with a Map.

---

29. An Address delivered at the Opening of the Medical Institution in Hope-street (Liverpool), on Wednesday, the 31st May, 1837. By John Rutter, M.D. President. Liverpool, 1837.

---

30. The Theory of Electric Repulsion examined in a Series of Experiments, &c. By Charles Hales. London. 8vo. Stitched. Pp. 22.

---

For the following Reports we are indebted to the kind politeness of Mr. Blizard Curling, of the London Hospital, a gentleman whose zeal for the advancement of medical science we have had several gratifying opportunities of noticing.

1. Institut Royal de France. Académie Royale des Sciences. Rapport sur le Concours du Grand Prix de Chirurgie. Diffomités du Systéme Osseux.

2. Rapport sur les Prix de Médecine et de Chirurgie, de l'Année, 1835. Par M. Serres, &c.

3. Annonce des Prix décernés par l'Académie des Sciences pour l'Année, 1836.

These Reports arrived too late for notice in the present Number. They will be brought before our readers in our next.

---

32. The Nature and Treatment of Diseases of the Ear. By Dr. Wm Kramer. Second Edition, translated from the German, by Jas. Risdon Bennett, M.D. 8vo, pp. 306, with plate. Longman & Co. Sept. 1837.

---

33. A Medico-Legal Treatise on Homicide by external Violence; with an Account of the Circumstances which modify the Medico Legal Characters of Injuries, &c. By Alex. Watson, Esq. 8vo. Maclachlan and Co. Sept. 1837.

### INDEX.

A.

Abdominal parietes, delivery of a fœtus through the ........ 492
Abdominal tumor, case of.......... 533
Abscess, small, opening a .......... 280
Abscess, large, opening a .......... 281
Abscess, deep, opening a .......... 282
Abuses of vital statistics .......... 185
Acanthus mollis.................. 148
Aconite ...................... 149
Acoustic nerve, deafness from compression of .................. 134
Acoustic nerve, deafness from palsy of 134
Action of certain medicines on the heart ...................... 526
Acupuncturation, cure of hernia by.. 561
Addison on diagnosis of pneumonia.. 37
Adhesion of the pericardium, case of 244
Agglutination of the os uteri, difficult parturition from.............. 235
Alibert, keloides of............... 326
Alvine concretions, Dr. Monro on .. 138
Alvine concretions, chemical analysis of........................ 141
Alvine concretions, causes of ...... 142
Alvine concretions, treatment of.... 144
America, medicine in............... 482
America and Ireland, fever in ...... 553
American Cyclopædia of Practical Medicine ...................... 129
Amphibia, nervous system of the.... 108
Amputation .................... 459
Amputation, unnecessary, caution against...................... 459
Amputation during suppuration .... 460
Amputation, preparations for ...... 461
Amputation, apparatus for ........ 461
Amputation, compression of artery for 462
Amputation at the hip-joint ....549, 550
Amputation, cases of.............. 550
Amputation at the knee-joint ...... 551
Amputation *in* the knee-joint ...... 551
Amputations of Dupuytren ........ 463
Anatomie du système dentaire ...... 155
Anatomie descriptive. By Cruveilhier 155
Anatomical plates, Quain's ........ 156
Anatomical plates, Bourgery's ...... 156
Anatomy of the nervous system, Mr. Anderson on the.............. 83
Anatomy of the lymphatic system .. 298
Anatomy and Physiology, Cyclopædia of...................... 154, 464
Anatomy of the skin ..........308, 320
Anderson on the anatomy of the nervous system .................. 83
Aneurysm cured by operation ...... 482
Annan on dislocations ............ 487
Annesley, Mr., reply of, to Mr. Twining.......................... 289
Anomalies de l'organization ........ 83
Anus, imperforate, case of ........ 230
Apothecary in Germany .......... 474
Appendix cæci vermiformis, inflammation of the .................. 23
Aqueous humor, posterior chamber of the .......................... 464
Arsenious acid, poisoning by ...... 263
Artillerymen, wounds incidental to.. 198
Ascites, cure of .................. 535
Assafœtida, action of, on the heart .. 526
Association, British, proceedings of.. 569
Asthma treated endermically ...... 525
Atrophy of bone ................ 6
Atrophy of old age................ 10
Atrophy of the heart's valves ...... 27

B.

Baden Baden .................... 423
Bad-Gastein .................... 427
Basis cranii, tumors at the ........ 265
Basis of the brain, tumors at the .... 265
Baudelocque on diseases of children.. 530
Bell's history of British quadrupeds.. 468
Bellis perennis .................. 147
Bibliographical record ............ 587
Bigger, Dr. on transplanting the cornea 477
Birmingham, prevalence of tape-worm in.......................... 285
Black expectoration, Dr. Thomson on 41
Bladder, injury to the neck of the .. 256
Bladder, sounding the ............ 259
Blennogenous apparatus .......... 316
Blood-letting .................... 418
Bone, atrophy of ................ 6
Bones, affections of the............ 3
Botany, Mr. Lindley on .......... 442
Bouillaud on fever................ 209
Bouillaud on typhoid fever ........ 450
Brain, Mayo on the state of the, in sleep ........................ 183
Brain, case of softening of the ...... 243
Brain, tumors at the basis of the.... 265
Brain, lesions of the .............. 271
Bracts .......................... 447
Breschet sur la Peau .............. 155
Breschet's Système Lymphatique .... 297
British flora medica .............. 145
British birds, history of, by Yarrell .. 468
British quadrupeds, history of ...... 468
British Association, proceedings of .. 569
Brodie, Sir B., on nervous affections 68

Bromide of potassium in diseases of the spleen ..................... 370
Bruit du diable .................... 186
Burne on inflammation, &c. of the cæcum ...................... 19

C.

Cæcum, Dr. Burne on inflammation, &c. of the ...................... 19
Calamine, constituents of .......... 187
Calculi in the kidney.............. 263
Calculus, size of the .............. 261
Calyx ........................... 448
Camphor, action of, on the heart.... 526
Cancer of the uterus treated endermically .......................... 524
Cancrum oris of children .......... 527
Carcinomatous growth from the cornea 276
Carlsbad ........................ 428
Carotid, right, aneurysm of the root of the ......................... 482
Carpus, dislocation of the, backwards 248
Cellular membrane, tumors of the .. 327
Cephalæa, remarkable case of ...... 200
Cerebral mass, weight of, compared with the intelligence ............ 507
Chancre ......................... 397
Chancre, use of caustic for ........ 399
Children, cancrum oris of .......... 527
Children, diseases of .............. 530
Chlorine, curative properties of .... 240
Chromatogenous apparatus ........ 317
Chronic pleuritis, effects of ........ 31
Circulatory organs, changes in the .. 97
Clavicle, osteo-sarcoma of the ...... 341
Clinical facts ..................... 280
Clinique Medicale. Par M. Bouillaud 450
Club-foot, cure of, by division of the tendon-Achillis ............499, 546
Colchicum, Dr. Lewins on.......... 174
Cold, Sir H. Halford on effects of.... 172
Colles on the venereal disease ...... 379
Congenital enlargement of the tongue 491
Contagion of glanders ............ 500
Copland's Dictionary of Practical Medicine ........................ 129
Cornea, carcinomatous growth from 276
Cornea, ossification of the.......... 277
Cornea, vascular prominence of the.. 277
Cornea, transplanting the, for blindness 477
Cough ending in emphysema of the body ........................ 188
Craigie on the practice of physic.... 403
Creosote, Sir F. Smith on ........ 177
Croup, cases of .................. 225
Cummin, Dr , death of ............ 288
Curative properties of chlorine...... 240
Cutaneous apparatus of inhalation .. 314
Cuvier sur l'Anatomie comparée .... 155
Cyclopædia of Practical Surgery 129, 459
Cyclopædia of Anatomy & Physiology 154, 464

D.

Deaf and dumb, language of gesture in the ........................ 245
Deafness, remedies for ............ 136
Deafness, causes of................ 130
Deafness from affections of the external ear..................... 130
Deafness and dumbness............ 136
Delirium, treatment of ............ 561
Denny on puerperal convulsions .... 485
Depletion for puerperal convulsions.. 485
Dermoid tumors.................. 325
Desgenettes, bibliographical sketch of 204
Devergie on syphilis .............. 379
Diabetes mellitus, Mr. M'Gregor on.. 188
Diabetes mellitus, Mr. Snowden on .. 188
Diabetes, Mr. Leach on lytta in .... 188
Diaphragmatic hernia, case of ...... 30
Diapnogenous apparatus .......... 313
Dictionary of Practical Medicine, by Copland ........................ 129
Difficult parturition, case of........ 234
Difficult parturition, from agglutination of the os uteri.............. 235
Digitalis, action of, on the heart.... 527
Diseases, general registration of .... 291
Diseases of Jersey ................ 377
Diseases of the Sandwich Isles ...... 493
Dislocation of the carpus backwards 248
Dislocation of the shoulders, reduction of .......................... 545
Dislocation of both thighs, simultaneous ........................ 546
Dissection wounds, Mr. Stafford on.. 351
Dothinenteritis .................. 553
Dupuytren, bibliographical sketch of 201
Dupuytren, his amputations........ 463

E.

Economy of health, Dr. J. Johnson on 79
Egra ............................ 434
Elements of medicine. By Dr. Williams 345
Eiloides ......................... 327
Emphysema of the body, cough ending in.......................... 188
Empyema cured by paracentesis .... 292
Ems.............................. 440
Encysted muscular melanosis ...... 329
Endermic and inoculative mode of treatment ...................... 524
Epidermoid tumors .............. 325
Epigastric region, violent pulsations in the ........................ 480
Erysipelas, M. Bouillaud on ........ 211
Erysipelas, poison of .............. 364
Erysipelas, infectiousness of........ 365
Erysipelas, contagiousness of ...... 367
Esculent mushroom, Edwards on poisoning with .................... 498
Etiology .......................... 404
Eustachian tube, deafness from diseases of ...................... 132

Eyeball, malignant affection of the .. 278
Existence of three mammæ in a man 505
Existence of four mammæ in a woman 505
Exostosis, maxillary, of the spongy texture .................... 337

F.

Factory morals, reformation of .... 284
Femoral aneurysm................ 482
Fever, M. Bouillaud on............ 209
Fever, puerperal, cases illustrative of 230
Fever in America and Ireland ...... 553
Fistula in perinæo ................ 258
Flora medica, British.............. 145
Fœtus, delivery of a, through the abdominal parietes................ 492
Fractured bones, reparation of...... 265
Frænum, perforating ulcer of the.... 401
Franzenbad, mud-baths of........... 434
Fright, insanity cured by .......... 540
Fumaria officinalis................ 147
Fungus hæmatodes, case of ........ 331

G.

Galvanism for tic douloureux ...... 190
Gangrene........................ 412
Gangrene of the leg, spontaneous.... 552
Gangrene of the lungs ............ 554
Gangrenous stomatitis ............ 529
Gastralgia, Dr. Lombard on ........ 532
Gastro-enterite, Louis on .......... 450
Gastro-enterite and diarrhœa, treatment of ...................... 560
Gastro-enteritis, case of .......... 273
Gastro-malacia .................. 213
Geary on typhoid fever ............ 557
Geneva, value of life in............ 184
Gerhard, Dr. on typhus fever ...... 553
Germany, Dr. Granville on the spas of 422
Germany, state of pharmacy in...... 473
Glanders, supposed, in the human subject........................ 246
Glanders in the human subject...... 359
Glanders, contagion of ............ 500
Glanders in man.................. 518
Glanders, chronic form of.......... 521
Glanders, gangrenous form of ...... 523
Glasgow Infirmary, expense of patients in.......................... 282
Grand climacteric ................ 79
Granville on the spas of Germany .. 422
Graves on the pathology of phlegmasia dolens...................... 501
Green on diseases of the skin ...... 156
Guy's Hospital reports ..........1, 249

H.

Hæmoptysis, sudden and fatal ... .. 585
Hæmorrhagic diathesis, cases of .... 540
Hæmorrhage, spontaneous, from the legs .......................... 541
Halford, Sir H., on the effects of cold 172
Hall on the practice of medicine .... 403
Hamerton's case of tetanus ........ 48
Hart's case of hernia pericardii...... 476
Hastings', Dr., address to the Worcestershire Natural History Society .. 158
Headland's oration................ 150
Health, Dr. J. Johnson on economy of 79
Heart's valves, atrophy of the ...... 27
Heart, Lees on wounds of the ...... 193
Heart, safety-valve of the.......... 265
Heart, Wardrop on diseases of the .. 469
Heart, action of certain medicines on the .......................... 526
Hermaphrodism.................. 114
Hermaphrodism, complex.......... 127
Hernia, stricture in .............. 281
Hernia pericardii, case of ........ 476
Hernia, cure of, by acupuncturation 551
Hip-joint, amputation at the ...... 549
History of British quadrupeds, by Bell 468
Hooper on the climate, &c. of Jersey 377
Hospital medicine and surgery...... 241
Hospitals, statistical report of ...... 539
Hudson on typhus fever............ 559
Hudson on the cause of tympanitic sound on percussion over an inflamed lung.................... 561
Human pathology, outlines of ...... 1
Human subject, supposed glanders in 246
Human subject, glanders in the .... 359
Hunter, John, works of ........153, 379
Hydrocele treated with iodine ...... 197
Hydrocephalus acutus, remarks on .. 219
Hyperémie cadaverique............ 348
Hypertrophy of the mammary gland.. 179
Hysteria, pathology of ............ 69
Hysteria, treatment of ............ 72

I.

Idiopathic tetanus and trismus ...... 227
Imperforate anus ................ 230
Increase of suicides in Paris ........ 211
India, medicine in ................ 196
Indolent pneumonia, tartar-emetic in 561
Inflammation of the spinal marrow .. 206
Inflammation, Dr. Yelloly on........ 345
Inflammation, causes of .......... 409
Inflammation, signs of ............ 410
Inflammation, nature of............ 414
Inflammation, modifications of...... 415
Inflammation, treatment of ........ 417
Inhalation, cutaneous apparatus of .. 314
Insane, treatment of the .......... 238
Insanity, causes of................ 538
Insanity among semstresses, frequency of.......................... 538
Insanity, cure of, by fright ........ 540
Intermittent fever and neuralgia .... 209
Intermittent fever treated endermically 524
Internal iliac, successful ligature of the 490
Intestines, lizards vomited from the.. 541
Iodine, treatment of hydrocele with.. 197
Irish medical charities bill.......... 285
Irritable ophthalmia .............. 276
Ischiatic notches, dislocation into the 488

J.

Jeaffreson's, Mr., case of successful removal of ovarian tumor........ 47
Jersey, climate and diseases of...... 377
Johnson, Dr. J., on economy of health 79
Johnson's, Dr. J., case of periodical monomania.................... 161
Johnson, Dr. J., on the railroad steamer ........................ 589

K.

Keloides ........................ 326
Key, Mr. Aston, on lithotrity ...... 250
Kidney, calculi in the ............ 263
Kidney with two ureters, case of.... 503
Kissengen ...................... 437

L.

Lallemand on spermatorrhea........ 239
Language of gesture in the deaf and dumb ........................ 245
Leach on lytta in diabetes.......... 188
Lead-colic, successful treatment of .. 284
Lees on wounds of the heart........ 193
Leg, spontaneous gangrene of the .. 552
Legs, tubercular sycosis of the...... 245
Lendrick on the nitro-muriatic acid bath........................... 179
Lepoides ........................ 325
Lewins on colchicum.............. 174
Lesions of the spinal chord ........ 270
Lesions of the brain .............. 271
Liebenstein...................... 437
Life, value of, in Geneva .......... 184
Lindley on botany ................ 442
List of original papers ............ 293
Liston on tumors of the mouth and jaws ........................ 10
Lithotomy ...................... 252
Lithotomy, dangers of ............ 254
Lithotrity, Mr. Aston Key on ...... 250
Little on pulmonary consumption .. 151
Lizards vomited from the intestines.. 541
Lobelia inflata, varieties of the...... 192
Lombard, Dr., on gastralgia........ 532
London Missionary Society ........ 290
Loss of memory .................. 372
Louis on gastro-enterite .......... 450
Lunatic hospitals, report on........ 535
Lunatics, table of ages of .......... 537
Lunatics, mortality of ............ 537
Lunatics, causes of death of ........ 539
Lymphatic system, anatomy of the .. 298
Lynn, Mr. death of................ 586

M.

M'Gregor on diabetes mellitus...... 189
Malcolmson on solitary confinement 170
Malignant disease of the stomach.... 176
Mammæ, existence of three, in a man 505
Mammary gland, Dr. Fingerhutt on hypertrophy of ................ 179
Marienbad ...................... 432
Maxillary exostosis of the spongy texture............................ 337
Mayo's Outlines of Human Pathology 1
Mayo on the philosophy of living.... 152
Measles, Dr. Williams on the poison of 361
Medical charities bill, Irish ........ 285
Medical education, Napoleon on .... 496
Medicine in India ................ 196
Medicine, elements of, by Dr. Williams 345
Medicine in America .............. 482
Medico-chirurgical transactions .... 1
Medico-chirurgical transactions .... 345
Melanotic growth from the semilunar membrane ...................... 277
Memory, loss of.................. 372
Mercury in the venereal disease .... 379
Modus agendi of poisons .......... 49
Monro on alvine concretions ...... 138
Morbid poisons, Dr. Williams on.... 49
Morbid poisons, effects of.......... 351
Morgue, statistical report from the .. 191
Mouth and jaws, tumors of the .... 10
Mower, Mr., death of.............. 287
Mucous matter, source of the ...... 316
Murray's Northern Flora .......... 190
Muscular melanosis, encysted ...... 329
Muscular tumors ................ 328

N.

Napoleon on medical education .... 496
National vaccine establishment, report of the ........................ 497
Navan fever hospital ............ 559
Necessity of scientific associations .. 158
Nervous affections, Sir B. Brodie on.. 68
Nitrate of silver in large doses ...... 172
Nitrate of silver for gonorrhœa of women.......................... 182
Nitro-muriatic acid bath, Dr. Lendrick on the use of the .............. 179
Northern Flora. By Dr. Murray .. 190
Nosogeny ...................... 405
Nosography .................... 406
Nosology........................ 405

O.

Obituary ....................287, 590
Operation, cure of aneurysm by .... 482
Ophthalmia, irritable.............. 276
Opium, use of, for ulcers .......... 467
Organic anomalies, cases of ........ 502
Organization, anomalies de l' ...... 83
Origin of the motor portion of the fifth nerve .......................... 465
Original papers, list of ............ 293
Osseous tumors .................. 336
Ossification of the cornea .......... 277
Osteo-sarcoma .................. 338
Osteo-sarcoma, origin of .......... 340
Osteo-sarcoma of the clavicle ...... 341
Ovarian tumor, successful removal of 47

P.

Paracentesis, cure of empyema by.. 292
Paralysis, cases of ................ 270
Paris, encrease of suicides in ...... 211
Parturition, difficult, case of........ 234

Pathology of pulmonary tubercles.... 32
Pathology of hysteria.............. 69
Pathology .................... 407
Pathology of the skin.......... .... 321
Peau, structure de la. Par M. Breschet 297
Pericardium, adhesion of the........ 244
Perineo, fistula in ................ 258
Periodical monomania, case of, by J. Johnson ........... .......... 160
Periosteal tumors ................ 335
Peritonitis .................... 257
Petreguin, Dr. on organic anomalies.. 502
Pharmacy, state of, in Germany .... 473
Philosophie anatomique............ 83
Philosophy of living, Dr. Ticknor on.. 152
Philosophy of living, Mr. Mayo on .. 152
Phlegmasia dolens, pathology of .... 500
Phlegmasia dolens, treatment of .... 501
Phloridzine, preparation of ........ 495
Phrenology, Dr. Sewall on ........ 159
Physiologie vegetale. Par M. Raspail 442
Pied-equin, case of................ 546
Plethora, deafness from... ........ 135
Pleurodinia, treatment of .......... 285
Plumbe on diseases of the skin...... 156
Pneumonia, Addison on diagnosis of.. 37
Poison of scarlatina .............. 59
Poison of measles ................ 361
Poison of erysipelas .............. 364
Poisoning by arsenious acid ........ 263
Polygala senega, action of, on the heart 527
Polypus nasi, gelatinous, spontaneous cure of a........ ............ 500
Population of the Sandwich Isles .... 495
Potassium, bromide of, in diseases of the spleen .................. 370
Practical medicine, American cyclopædia of .................... 129
Practical surgery, cyclopædia of .... 129
Practical surgery, cyclopædia of .... 459
Practice of medicine, Dr. Hall on the 403
Practice of physic, Dr. Craigie on the 403
Preparation of phloridzine.......... 495
Progress of syphilis .............. 393
Provincial Med. and Surg. Association, transactions of ................ 345
Psychological physiology .......... 507
Puerperal fever, cases illustrative of.. 230
Puerperal convulsions, Dr. Denny on.. 485
Puerperal convulsions, depletion for.. 485
Puerperal convulsions, case of ...... 486
Pullna ........................ 436
Pulmonary tubercles, pathology of .. 32
Pulmonary consumption, Mr. Little on 151
Pulsations, violent, in the epigastric region ...................... 480
Purgatives, treatment of typhoid fever by .......................... 510

R

Railroad steamer, by Dr. J. Johnson.. 589
Ramollissement of the spinal marrow 283
Rasori, death of .................. 586
Raspail on vegetable physiology .... 442
Recent pathological transactions .... 1
Recurrent nerve, why recurrent .... 282
Reformation of factory morals...... 284
Registration of diseases............ 291
Remarks on hydrocephalus acutus .. 219
Removal of the superior maxillary .. 18
Reparation after simple fracture .... 3
Reparation of fractured bones ...... 265
Report of the natural vaccine establishment .................. .... 497
Report on lunatic hospitals ....... 536
Report of patients in St. George's Hospital ...................... 562
Rete mirabile in the sloth.......... 463
Retroversio uteri ................ 173
Rheumatism treated endermically... 525

S.

St. George's Hospital, report from .. 562
Saint Hilaire sur la philosophie anatomique ...................... 83
Sandwich isles, diseases of the ...... 493
Sandwich isles, climate of the ...... 494
Sandwich isles, venereal disease in the 494
Sandwich isles, population of the.... 495
Scarlatina, poison of .............. 59
Scarlatina, pathology of............ 60
Scarlatina, gravior ................ 61
Scarlatina, mitior ....... . ...... 66
Scarlatina, treatment of............ 63
Scars in the neck, how to prevent .. 280
Schlangenbad .................... 439
Schwalbach ......... ............ 440
Scientific associations, necessity of .. 158
Sclerotica, bulging of the .......... 278
Scirrhous muscular tumor ........ 329
Section of the sterno-mastoid muscle 548
Semilunar membrane, melanotic growth from .................. 277
Senile hydro-nephrosis ............ 503
Seven half-crowns swallowed ...... 187
Sewall on phrenology.............. 159
Shoulder, reduction of dislocation of the . ...................... 545
Simultaneous dislocation of both thighs 546
Skey on ulcers .................. 466
Skin, Mr. Plumbe on diseases of the 156
Skin, Dr. Green on diseases of the .. 156
Skin, anatomy of the ..........308, 320
Skin, pathology of the ............ 321
Sloth, rete mirabile in the.......... 463
Smith, Sir F. on creosote .......... 177
Snowden on diabetes mellitus ...... 188
Softening of the brain, case of...... 243
Solitary confinement, Mr. Malcomson on .............. ........ 170
Spas of Germany, Dr. Granville on.. 422
Spasmodic affection of the thumb in writing, &c.................... 226
Spermatorrhea, Lallemand on ...... 239

Spina bifida, case of .............. 228
Spinal marrow, inflammation of .... 206
Spinal marrow, ramollissement of the 283
Spinal chord, lesions of the ........ 270
Spleen, bromide of potassium in diseases of the .................. 370
Spontaneous gagrene of the leg...... 552
Stafford on dissection wounds ...... 351
Stamens, transformation of ........ 449
Statistical report from the Morgue .. 191
Statistical report of hospitals ...... 539
Sterno-mastoid muscle, section of the 548
Stomach, malignant disease of the .. 176
Stream of cold water in wounds .... 542
Stricture in hernia................ 282
Structure de la peu. Par M. Breschet 297
Substitute for liquor opii sedativus .. 498
Successful ligature of internal iliac .. 490
Sudden and fatal hæmoptysis........ 585
Sudoriferous canals ............... 313
Supposed new views of dislocations.. 487
Syphilis, M. Devergie on ........... 379
Syphilis, natural history of ........ 390
Syphilis, progress of ............... 393
Système Lymphatique. Par M. Breschet 297
T.
Tape worm in Birmingham, prevalence of ....................... 285
Tape-worm, case of................. 567
Tendo-achillis, division of the, for club-foot ....................... 546
Tendons, tumors of the ............ 332
Testicles, persistence of the, within the abdomen .................. 504
Tetanus, successful treatment of .... 48
Therapeutics ...................... 407
Thomson on black expectoration .... 41
Thoracic inflammation masked ...... 181
Thoracic viscera, lateral transposition of the ........................ 506
Thorax, vicarious menstruation from the skin of the ................ 497
Thumb, spasmodic affection of, in writing, &c...................... 226
Tic douloureux, relieved by galvanism 190
Ticknor on the philosophy of living.. 152
Toeplitz .......................... 436
Tongue, treatment of a wound of the 199
Tongue, congenital enlargement of the 491
Transplanting the cornea for blindness 477
Transposition of the viscera ........ 584
Transactions of the Provincial Med. and Surg. Association .......... 1
Transatlantic snuff chewing ........ 196
Treatment of the insane............ 238
Tubercular sycosis of the legs ...... 245
Tumors at the basis of the brain .... 265
Tumors at the basis cranii.......... 265
Tumors, Dr. Warren on............ 322
Tumors, examination of............ 324
Tumors of the cellular membrane.... 327
Tumors of the tendons ............ 332
Tympanitic sound, cause of, on percussion over an inflamed lung .... 561
Tympanum, diseases of the membrane of the ......................... 131
Tympanum, inflammation of the cavity of.............................. 133
Typhoid poison .................... 50
Typhoid fever, use of purgatives in.. 510
Typhoid fever, depletion in ........ 513
Typhoid fever, contagion of ........ 555
Typhoid fever, pathological anatomy of 556
Typhoid fever, Dr. Geary on ........ 557
Typhoid fever, chloride of soda in .. 561
Typhus, treatment of............... 55
Typhus fever ...................... 209
Typhus abdominalis, use of chlorine in 240
Typhus fever, M. Bouillaud on ...... 450
Typhus fever, M. Louis on ........ 450
Typhus fever, treatment of ........ 454
Typhus fever, Dr. Gerhard on ...... 553
Typhus fever, treatment of. ........ 559
U.
Ulcers, Mr. Skey on ............... 466
Ulcers, use of opium for .......... 467
Urachus, persistence of the cavity of the ............................ 504
Urinary bladder, case of worms in the 495
Uterus, cancer of the, treated endermically.......................... 524
V.
Value of life in Geneva............ 184
Vance, Mr. death of .............. 288
Varicose aneurysm, case of ....... 24
Varietes of the lobelia inflata ...... 192
Vascular prominence of the cornea.. 277
Venereal disease, use of mercury in.. 379
Venereal disease, Dr. Colles on the .. 379
Vicarious menstruation from the skin of thorax......................... 497
Viscera, transposition of the........ 584
Vital statistics, abuses of .......... 185
W.
Wardrop on diseases of the heart.... 469
Warren on Tumors ............... 322
Weisbaden ....................... 438
Wildbad ......................... 424
Williams on morbid poisons ........ 49
Williams on the elements of medicine 345
Williams on the poison of measles .. 361
Worcester Infirmary, report from.... 278
Worcestershire Natural History Society, address to, by Dr. Hastings.. 158
Works of John Hunter............. 379
Works of John Hunter ............ 153
Worms in the urinary bladder ...... 495
Wounds, stream of cold water in.... 542
Wry neck, case of ............... 548
Y.
Yarrell's history of British birds . .. 468
Yelloly on inflammation .......... 345

